# A few of my favorite things: circumscribed interests in autism are not accompanied by increased attentional salience on a personalized selective attention task

**DOI:** 10.1186/s13229-017-0132-1

**Published:** 2017-04-12

**Authors:** Owen E. Parsons, Andrew P. Bayliss, Anna Remington

**Affiliations:** 1 Autism Research Centre, Department of Psychiatry, University of Cambridge, Cambridgeshire, UK; 2 School of Psychology, University of East Anglia, Norwich, UK; 3 Centre for Research in Autism and Education, UCL Institute of Education, University College London, 55-59 Gordon Square, London, WC1H 0NU UK

**Keywords:** Autism, Circumscribed interests, Special interests, Attention, Perception

## Abstract

**Background:**

Autistic individuals commonly show circumscribed or “special” interests: areas of obsessive interest in a specific category. The present study investigated what impact these interests have on attention, an aspect of autistic cognition often reported as altered. In neurotypical individuals, interest and expertise have been shown to result in an automatic attentional priority for related items. Here, we examine whether this change in salience is also seen in autism.

**Methods:**

Adolescents and young adults with and without autism performed a personalized selective attention task assessing the level of attentional priority afforded to images related to the participant’s specific interests. In addition, participants performed a similar task with generic images in order to isolate any effects of interest and expertise. Crucially, all autistic and non-autistic individuals recruited for this study held a strong passion or interest. As such, any differences in attention could not be solely attributed to differing prevalence of interests in the two groups. In both tasks, participants were asked to perform a central target-detection task while ignoring irrelevant distractors (related or unrelated to their interests). The level of distractor interference under various task conditions was taken as an indication of attentional priority.

**Results:**

Neurotypical individuals showed the predicted attentional priority for the circumscribed interest images but not generic items, reflecting the impact of their interest and expertise. Contrary to predictions, autistic individuals did not show this priority: processing the interest-related stimuli only when task demands were low. Attention to images unrelated to circumscribed interests was equivalent in the two groups.

**Conclusions:**

These results suggest that despite autistic individuals holding an intense interest in a particular class of stimuli, there may be a reduced impact of this prior experience and expertise on attentional processing. The implications of this absence of automatic priority are discussed in terms of the behaviors associated with the condition.

**Electronic supplementary material:**

The online version of this article (doi:10.1186/s13229-017-0132-1) contains supplementary material, which is available to authorized users.

## Background

An intriguing feature of autism is the presence of areas of intense interest in the vast majority of individuals with the condition. Here, we examine how these interests are reflected in autistic individuals’ perceptual processes, and what implications this has for our understanding of autistic cognition.

Autism is a developmental condition thought to affect around 1% of the UK population [1, 2]. While the severity and symptom profile can vary across individuals, autism is clinically defined by deficits in social communication and interaction, as well as the presence of restricted, repetitive behavior, interests or activities (RRB) [3]. The latter comprises aspects such as stereotyped or repetitive movements and speech, insistence on sameness and altered sensory sensitivities. While these symptoms can be as problematic for autistic individuals as the better-known social features of the condition [4], RRBs can also manifest themselves as strong passions or specific talents [5–7]. Referred to as circumscribed—or special—interests, these passions generally involve an intense level of interest in a narrow topic [8].

The evidence also points toward regular themes within the topics of interest of individuals with ASD. Topics of interests in autism tend to fall in non-social domains and focus on science and technology-related topics, which are not so popular among the typically developed population. Examples include memorizing historical dates and the periodic table and understanding how mechanisms work [9]. Individuals with autism show a strong tendency to read thoroughly around such topics, and often focus on rote memorization of facts relating to their circumscribed interest [10].

The proportion of individuals with autism that possess a circumscribed interest is thought to be very high, with research suggesting that 75% of children and 88% of adolescents report a circumscribed interest or passion [11]. Despite their prevalence, circumscribed interests are rarely investigated: there is a disproportionally low volume of published research in the area in contrast to the other aspects of RRBs in autism. A search on PubMed, for example, reveals 36 items for “autism” and “circumscribed interests” versus over 550 for “autism” and “repetitive behaviors”. The disparity in volume of research on circumscribed interests compared with other areas is particularly striking when one considers the potential impact of special interests in educational and clinical environments. Anecdotal evidence from the autism community suggests that personal identity is often anchored in these interests and provides a focus for social interaction with caregivers and others. It has been suggested that special interests can be used as a reward to improve challenging behavior [12, 13] and also to increase social engagement with peers [14, 15]. Further, circumscribed interests have been successfully incorporated into cognitive behavioral therapies used to treat anxiety in children with autism [16, 17]. Conversely, circumscribed interests have been associated with disruption to schoolwork and social function [18, 19]. While it is not yet clear whether circumscribed interests should be regarded as a helpful or disruptive trait [20], their prevalence in autism suggests that they might offer an insight into autistic cognition. To that end, the present study explored the relationship between circumscribed interests and attention and perception in the condition. Specifically, we examined the impact of these interests on selective attention, a cognitive process widely reported to be atypical in autism.

Selective attention, the process by which people are able to attend and react to certain stimuli while ignoring others [21], is a core component of our ability to complete everyday tasks such as navigating through a crowd or holding a conversation. Whether or not a specific item is processed depends on its salience within the environment. This salience can be dictated by either top-down or bottom-up factors [22]. Bottom-up factors (generally innate and universal) refer to the extent to which a stimulus “pops out” from its surroundings, due to differences in low-level features such as color or shape [23]. For example, a red poppy in a green field of grass will have salience due to its contrasting color. On the other hand, top-down factors vary from person to person, tend to be more goal-directed, and increase the salience of stimuli that are relevant to one’s state or experiences [24]. An individual with a passion for bird watching might have his or her attention drawn toward a particular species of bird in a tree, while others would pay little attention to it. While bottom-up factors are generally innate and universal, top-down factors vary significantly from person to person and so an individual’s personal interests will influence perception in a top-down manner. One universal top-down influence in neurotypical individuals is the tendency to show a strong preference for social stimuli: with face stimuli being afforded attentional priority over other items. This attentional bias has been found in newborns [25] and continues throughout life.

Indeed, there appear to be neural regions dedicated to the processing of social stimuli (e.g., faces), with a large body of research revealing that the fusiform gyrus (FG) is strongly implemented in a range of face processing tasks [26–29]. In addition, it has been suggested that these areas can be recruited in response to expertise in other categories [30], with increased activation seen after the acquisition of expertise with novel objects [31]. Similarly, car experts showed higher activation in the right fusiform face area and occipital face area when exposed to pictures of cars, compared to pictures of birds, and bird experts showed the reverse effect [32].

This may have implications for autism. While an attentional bias toward faces has been robustly found in the neurotypical population, these effects do not appear to be present in autism. Individuals with autism have been shown to display a reduced preference for faces over objects [33, 34] as well as reduced activity in the FG during exposure to faces [35] and no automatic priority for attention [36]. The apparent absence of this specialization for social items raises two hypotheses: (1) the lack of social specialization in autism reflects the reduced interest in social items, while the expertise in circumscribed interest topics would lead to specialization and activations in the FG, or (2) there is reduced/absent specialization in response to any class of item (related or unrelated to personal interests). Lending initial support for the former hypothesis, a case study of an individual with autism found increased activity in the FG when presented with images of “Digimon” cartoon characters—his special interest—but not when shown human faces [37]. Likewise, recent work with a group of autistic and non-autistic participants—both with interests—has shown more robust responses to interests in the FG for the autistic individuals [38]).

On a behavioral level, one way to explore the alternative hypotheses is to examine whether items related to circumscribed interests show automatic top-down priority for attention over other objects—similar to the way in which faces capture attention in typical individuals. Here, we use the Load Theory of Attention and Cognitive Control [39, 40] to investigate this. Load Theory asserts that the degree to which task-irrelevant stimuli are processed is determined by the perceptual load of a task (the amount of potentially task-relevant information). When perceptual load is high, exhausting processing capacity, irrelevant stimuli are ignored. Conversely, when perceptual load is low, spare processing capacity remains, and this spills over resulting in additional irrelevant processing. The level of interference of task irrelevant stimuli and distractors decreases with increasing perceptual load of the central task (e.g., [41–43]). However, this modulation of attention by load is not seen for social stimuli or stimuli related to areas of expertise—both of which appear to capture attention irrespective of load level. When human faces were presented as distractors, offset from a central target categorization task, their interference effect remained constant—despite increasing perceptual load (additional non-target elements) in the central task [44]. This result supports previous findings that human faces have a special saliency and are processed in an automatic and mandatory fashion [26, 45]. Similarly, in expert musicians, irrelevant pictures of musical instruments were processed irrespective of load level [46]. This pattern was not seen in non-musicians, suggesting that salience is modified by expertise. As such, this framework can be used to establish the relative salience of a class of stimuli for each individual participant.

We have recently used this paradigm to examine whether the same pattern of results is seen when autistic individuals perform a task that involves social distractor images. Results indicated that the autistic adults processed the face distractors in conditions of low load but that this interference effect was eliminated under high load. Unlike in neurotypical adults, there seemed to be no “special salience” for the social items: those in the autism group were able to ignore the irrelevant face stimuli at higher levels of perceptual load while the non-autistic controls remained distracted by them. On the corresponding non-social task, the two groups performed similarly: the distractor interference effect decreased as load increased [36]. The pattern observed for items related to circumscribed interests in autism may offer a resolution to the two opposing hypothesis outlined above. Given the high level of expertise, it seemed likely that circumscribed interest items would show an automatic priority for attention in autistic individuals. However, if no such priority is seen, it may suggest an absence of specialization to any class of stimuli based on interest and expertise.

Previous literature on attention to circumscribed interests in autism is scarce. The few existing studies have tended to employ preferential looking paradigms (using eye-tracking techniques). Sasson and colleagues carried out a series of studies that used a passive-viewing paradigm to assess patterns of attention to non-social stimuli using a mix of objects either related (e.g., trains, planes, automobiles, etc.) or unrelated (e.g., plants) to typical circumscribed interest topics in autism. Autistic children showed a higher degree of exploration and fixation [47] and increased self-reported preference (compared to non-autistic controls) [48] for the items related to typical circumscribed interests. When faces were presented alongside non-social stimuli, the autism group showed typical attention to faces when non-social items were not connected to their interests but showed significantly lower attention to faces when they were presented alongside objects of interest [49].

Using a generalized stimulus set based on common categories of interest, these studies offer preliminary evidence that objects of interest have a special salience in autism. Yet, despite having similar themes, we would expect there to be a large amount of heterogeneity in the interests of the autism group. Therefore, while these tasks may be able to detect overall group differences, it is likely that some of the autism group will not possess a genuine interest in the stimuli, leading to unnecessary variance in the data and reduced validity of the task. Secondly, as a disproportionately high number of autistic individuals have strong interests in comparison to the neurotypical population, the effects found could in part be attributed to group differences in interest strength or presence of any strong interest as opposed to being attentional differences unique to autism.

In the present study, we have developed a personalized task that allows the systematic investigation of attentional priority for each participant. Furthermore, we have included a comparison group that was comprised of neurotypical individuals with interests and passions. In addition, by using the Load Theory paradigm, we can specifically look at whether individuals are able to ignore stimuli related to their circumscribed interest when they have been explicitly instructed not to attend to them. In such tasks, neurotypical participants have been shown to automatically prioritize items related to their interests and expertise, even at the expense of central-task performance. These tasks have not yet been conducted with autistic individuals and are distinct from the free-viewing paradigms used previously to investigate circumscribed interests, which have only assessed preference for high interest items in the absence of task demands. We predicted that, given all participants had a passion/interest, both groups would show processing of distractor images related to their interests under all levels of load. This would be in contrast to the processing of generic images, which would only be seen under low load conditions.

A greater understanding of the attentional priority given to circumscribed interests will offer insight into attentional specialization in autism. Further, awareness of exactly how circumscribed interests draw attention in autism may allow them to be more effectively integrated into behavioral and educational interventions.

## Methods

Two experiments were carried out to assess whether objects related to participants’ interests hold a different salience to generic objects. This was done by measuring the interference of both classes of stimuli on a selective attention task.

### Experimental stimuli

Stimuli were presented using the OpenSesame software package [50] on a 15” Dell laptop. Participants were positioned with a 60 cm viewing distance from the screen. An external keyboard was used to record button responses. During each trial, a target word was presented in the center of the screen. In experiment 1, individualized circumscribed interest words and distractor images were used. These were related to each participant’s individual interest (e.g., car manufacturer vs. motorbike manufacturer for an automobile enthusiast). No social items (faces) were included in the distractor images, to ensure that the results would not be confounded by social priority for attention in the non-autistic participants. For example, when the interest was Harry Potter the stimuli were Hogwarts School house logos, items of clothing, wands, etc. In experiment 2, the names of stringed or wind instruments and an associated image set (Wind: clarinet, horn, saxophone, trombone, trumpet, tuba; Stringed: banjo, bass, cello, guitar, harp, violin) were used.

**Fig. 1:**
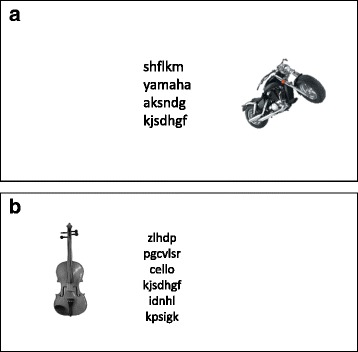
Example of stimuli used in experiment 1 (**a**) and Experiment 2 (**b**). **a** A congruent trial of set size 4. **b** A congruent trial of set size 6. For clarity, the examples are shown here without the gray background, and in grayscale (full-color images were used in the experimental tasks)

Participants were required to classify the word into one of two categories. A set of six words from each category was used throughout the task. The perceptual load of the task was varied by presenting additional non-words above and below the target word. There were four levels of load: target word presented alone (set size 1), target word and one non-word (set size 2), target word and three non-words (set size 4) or target word and five non-words (set size 6). All text was presented in size 23 sans-serif text in black against a gray background. In addition to the word lists, corresponding distractor images (in color) were also present in every trial. During each trial, a distractor image measuring 4.1° × 3.3° visual angles appeared alongside the word list. Distractor images were presented to the left or right of the word lists, 5° from the center of the screen (see Fig. 1). These images were either congruent (from the same category as the target word) or incongruent (alternative category to the target word). The task was split into eight blocks (two of each set size) of 32 trials. All trials were separated by the presentations of a central fixation cross for 500 ms. Condition presentation order, target position within the word list, and distractor condition were all counterbalanced. Response time (RT) and accuracy on each trial were recorded automatically by the OpenSesame software package. Though an indirect measure, previous research suggests that RT aligns with more explicit attention measures (e.g., awareness reports [51]) and is therefore considered appropriate for the current study.

### Procedure

Before starting the task, full-word lists were presented to participants to ensure that they were familiar with all items. Participants were instructed to locate the target word among the non-words and to indicate which category the word fell into with a button press. The response buttons used were the left and up arrows. Individualized stickers were placed on these keys for each participant, showing a letter to represent the category choice (e.g., “C” for cars or “M” for motorbikes). Participants were explicitly told to ignore the distractor images and to complete the task as fast as possible. RT (milliseconds from stimuli presentation) and accuracy were recorded for each trial. Before taking part in the main study, all participants completed a 16-trial practice block. Trials for all four set sizes appeared equally in the practice block to allow participants to experience all possible levels of perceptual load. Participants were given a short break and encouragement in between each block.

### Experiment 1

Experiment 1 examined whether an automatic priority for attention is seen for images related to autistic and non-autistic individuals’ specific interests. The categories used within the task were specifically related to the interests of each participant.

### Participants

Fifteen adolescents/young adults with a diagnosis of autism (aged 13–32 years) and 17 non-autistic neurotypical controls (aged 13–20 years) were recruited via schools, personal contacts and advertisements (see Table 1 for summary information). Sample size (accounting for expected attrition) was determined by previous research using similar paradigms [51]. IQ (all >80) was assessed using the Wechsler Abbreviated Scale for Intelligence (WASI; [52]). The reading component of the tasks restricted participation to those without intellectual impairment. Groups were matched on age and non-verbal IQ (using the Matrix Reasoning subtest of the WASI). All participants in the autism group had previously received a clinical diagnosis of an autism spectrum condition from an independent qualified clinician in accordance with the Diagnostic and Statistical Manual of Mental Disorders, Fourth or Fifth Edition [3, 53]. None of the participants had any other neurological condition. Diagnosis in autistic participants was confirmed by the researchers using Module 4 of the Autism Diagnostic Observation Scale (ADOS; [54]) and the Social Responsiveness Scale [55] was given to all control participants to check for the possibility of undiagnosed cases. Two individuals from the neurotypical group were excluded due to high scores on the Social Responsiveness Scale (SRS) (above “normal limits”, *T* score of >59). One individual from the autism group was excluded due to being unable to engage with the task correctly. *T* tests indicated that IQ was higher in the neurotypical group (*p* = .008), as was performance on the vocabulary subtest of the WASI (*p* < .001). There was no difference in WASI Matrix Reasoning scores (non-verbal subtest) or in the age of the two groups (both *p* values >.6).

**Table 1:** Descriptive statistics for each group in experiment 1

		Age	WASI		
		(years:months)	Vocabulary subtest	Matrix reasoning	Full scale IQ
					(2 subtests)
Autism (*n* = 14, all males)	Mean	19:04	46	55	101
	(SD)	(7:4)	7	11	12
	Range	13:0–32:6	35–58	36–80	84–128
Neurotypical (*n* = 15, all males)	Mean	18:04	64	56	118
	(SD)	(2:3)	5	9	10
	Range	13:9–20:8	52–72	37–68	99–136

We specifically recruited people who had a strong interest or passion (based on self report). We consider this to be a key strength of our experimental design (as discussed above); however, it undoubtedly made recruitment more challenging and limited the sample sizes. Once an individual was recruited, we spoke to them (or their teacher/parent in the case of younger participants) to determine what their passion was. There was some overlap in the interests of the two groups (Premier League Football; My Little Pony) and some interests that were unique to individuals in the autism (Magic, Disney Pixar films, trains, automobiles, various films/computer games, comic books, World War II, Pokémon) or neurotypical group (Harry Potter, Cricket, Guns, Gymnastics, Horses). A summary of the different individual interests included in the study can be seen in Table 2.

**Table 2:** Summary of different interests

Special interest/hobby	Diagnostic group (autism/neurotypical)
Magic	Autism
Disney Pixar films	Autism
Trains	Autism
Automobiles	Autism
Horror films	Autism
PC games	Autism
Comic books	Autism
Super Smash Bros	Autism
World War II	Autism
Films	Autism
Pokémon	Autism
Harry Potter	Neurotypical
Cricket	Neurotypical
Guns	Neurotypical
Gymnastics	Neurotypical
Horses	Neurotypical
Premier League Football	Both groups
My Little Pony	Both groups

### Participant Assessment of Significance of Special Interests and OccupatioNs (PASSION) questionnaire

In addition to the computer-based task, participants in experiment 1 were asked to complete a short questionnaire to assess the intensity of their interest in the item used on the experimental task (PASSION data is missing for three autistic individuals due to school absence during data collection). The Participant Assessment of Significance of Special Interests and OccupatioNs (PASSION) questionnaire was developed specifically for this study to allow us to assess the nature of the participants’ interest in the item used in their personalized task. The PASSION questionnaire was based on previous parental-report measures of special interest [8] (M. South, A. Klin, S. Ozonoff: The Yale Special Interests Interview, unpublished), and was a brief, self-report version that is more appropriate for adolescents and young adults. This measure was used to investigate whether there was a difference between our autistic participants and neurotypical controls in the self-reported interest regarding the item in question. A systematic difference in the level of interest in the two groups (e.g., less strong interests in the neurotypical group) might have undermined the validity of the experimental task results. The 15-item questionnaire was designed to assess different key aspects of a passion/interest including level of importance to the individuals, whether the interest interferes with daily functioning, level of expertise in interest, duration of interest and whether the individual possessed any other interests. All items, except for one asking participants to list any other interests they possessed, were scored on a Likert scale (Full details of the questionnaire can be found in the “Additional files” section (see Additional file 1).

Scores for each subscale (importance, interference, expertise, duration of interest, number of other interests) were calculated for each participant (15 neurotypical, 11 autistic). For full questionnaire, see the “Additional filesˮ section. Independent *T* tests showed no difference between the groups on any subscale (all *p* values >.39). On average, both groups indicated that the interest was very important, but they somewhat disagreed with the idea that the interest interfered with daily functioning (work, school, relationships, etc.). They felt that they knew a bit more than others who have the same interest, and their interests had generally lasted between 3 and 5 years. In both groups, the majority of participants had one or two additional important interests (see Table 3 for details).

**Table 3:** Average scores for each group on each subscale of the PASSION questionnaire (with SD in brackets)

	Autism	Neurotypical	Significance (*p*)
Mean number of interests	1.5 (1.8)	1.6 (1.6)	0.94
Mean importance score	3.9 (.39)	4.2 (.85)	0.37
Mean interference score	2.2 (1.1)	2.7 (1.2)	0.35
Mean expertise score	4.2 (.65)	4.4 (.40)	0.5

This is important, as it confirms that differences in the experimental task are not due to different levels of interest or expertise in the two groups

## Results

### Accuracy

Accuracy was high (>92%) for both groups (see Table 4). Mean proportion correct values for each participant, for each condition, in both groups were submitted to a mixed-factor ANOVA. There was a main effect of congruency (*F* (1, 27) = 5.15, *p* = .032, η_p_^2^ = 0.16): the accuracy rates were higher for the congruent trials; no other main effect or interaction approached significance (all *p* values >.2).

**Table 4:** Overall mean median RT (ms) and mean accuracy rates (proportion correct) and standard deviations (SD) for the two groups under congruent (cong.) and incongruent (incong.) distractor conditions at each set size in experiment 1

		Set size 1		Set size 2		Set size 4		Set size 6	
		cong.	incong.	cong.	incong.	cong.	incong.	cong.	incong.
Autism	RT	745	788	896	894	1226	1162	1410	1395
	(SD)	195	206	256	250	329	270	311	354
	Accuracy	0.97	0.93	0.95	0.92	0.93	0.93	0.93	0.92
	(SD)	0.05	0.05	0.05	0.08	0.12	0.07	0.1	0.13
Neurotypical	RT	643	685	703	766	883	912	1068	1129
	(SD)	77	81	99	77	138	137	178	204
	Accuracy	0.96	0.95	0.97	0.95	0.96	0.93	0.98	0.96
	(SD)	0.04	0.06	0.02	0.05	0.07	0.06	0.03	0.06

### Reaction time

Median reaction time (RT) for correct trials in each condition was calculated for each participant (see Table 3). The median was used, rather than the mean, to avoid extreme values (which may result from momentary lapses in concentration) disproportionately influencing the average. A mixed ANOVA was performed using diagnosis group as a between-subject factor and both perceptual load (set-size) and distractor congruency as the within-subject factors.

There was a significant main effect of set size, (*F* (3, 81) = 207.3, *p* < .001, η_p_^2^ = 0.89), congruency (*F* (1, 27) = 6.91, *p* = .014, η_p_^2^ = 0.20), and diagnostic group (*F* (1, 27) = 8.85, *p* = .006, η_p_^2^ = 0.25). This reflects the fact that RT, increased as the perceptual load of the task increased, was slower for incongruent than congruent trials (966 and 947 ms respectively) and that the autistic participants were slower than the neurotypical controls (ASD: *M* = 1064 ms; neurotypical: *M* = 848 ms). There was a significant interaction between set size and group (*F* (3, 81) = 9.03, *p* < .001, η_p_^2^ = 0.25), between congruency and group (*F* (1, 27) = 15.3, *p* = .001, η_p_^2^ = 0.36), and between set size and congruency (*F* (3, 81) = 3.06, *p* = .033, η_p_^2^ = 0.10). Inspection of the means suggests that the latter reflects the fact that the congruency effect declined as set size increased.

**Fig. 2:**
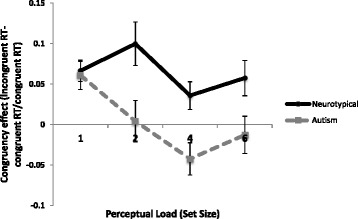
Congruency effects of items of interest for the two groups across all set sizes. Congruency effects (as a proportion of the baseline RT) were calculated for each person by subtracting average congruent trial RT from the average incongruent RT and dividing by congruent RT. This gives a proportionate measure that accounts for individual differences in RT. *Error bars* indicate the standard error of the mean

To further investigate the significant group interactions, repeated measure ANOVA was carried out for each group separately. The control group showed a stable significant congruency effect (*F* (1, 14) = 84.5, *p* < .001, η_p_^2^ = 0.86) that did not significantly reduce as the set size increased (no significant interaction, *p* = .49). Contrarily, the autism group showed no overall main effect of congruency (*p* > .5) but a significant interaction between set-size and congruency (*F* (3, 39) = 3.32, *p* = .03, η_p_^2^ = 0.20), i.e., there was a congruency effect at the lower set sizes, which declined as the load of the task increased (see Fig. 2). The absence of a congruency effect indicates that the distractor item is not being processed (i.e., a participant is not helped by a congruent picture or hindered by an incongruent one). As such, the significant interaction implies that the neurotypical individuals processed the distractors related to their items of interest at all levels of load, whereas the autistic individuals only processed them at the lowest level of load.

As the groups differed in IQ, this was added as a covariate and a repeated measure ANCOVA was run. All significant main effects and interactions remained, except for the main effect of group. This was no longer significant *F* (1, 26) = 1.54, *p* = .226, η_p_^2^ = 0.06 and suggests that group differences in overall RT were due to the lower IQ in the autism group.

### Experiment 2

The results from experiment 1 indicated that neurotypical individuals, but not autistic individuals, processed pictures of items related to their passions/interests at all levels of perceptual load. In order to confirm that the persistence of an interference effect in the control group was due to expertise, a second task using non-special interest objects was employed. This task, following that of Lavie and colleagues [44] used pictures and names of musical instruments, and we ensured that there were no expert musicians in either group.

### Participants

In total 14 participants with autism and 19 typically developing controls took part in the second experiment. Sample size was determined by previous research using similar paradigms [51]. Nine of the autistic participants and five controls were re-recruited from the first experiment. In these cases, there was a gap of several weeks between testing sessions, to avoid practice effects. All other participants were recruited specifically for the study using the same recruitment methods as experiment 1.

Participants were aged between 14 and 32 years old and all had IQs (assessed using the WASI) in the typical range (>80). As before, diagnosis was confirmed using the ADOS and the SRS was issued to all controls. Age and performance IQ were matched between the two groups. Newly recruited participants were also quizzed on their personal interests to ensure that they did not possess a particularly strong interest in musical instruments. Four individuals from the neurotypical group were excluded due to high SRS levels (above normal limits, *T* score >59). Details of the remaining 30 participants can be seen in Table 5. *T* tests revealed that the groups differed on performance on the vocabulary subtest of the WASI (*p* = .001) but were not significantly different on any other measure, including IQ (all *p* values >.1).

**Table 5:** Descriptive statistics for each group in experiment 2

		Age	WASI		
		(years:months)	Vocabulary subtest	Matrix reasoning	Full scale IQ
					(2 subtests)
Autism (*n* = 14, all males)	Mean	19:04	51	60	109
	(SD)	(7:0)	8	12	12
	Range	13:0–32:6	39–66	46–80	89–128
Neurotypical (*n* = 15, all males)	Mean	17:04	63	56	117
	(SD)	(2:4)	9	12	15
	Range	13:0–20:9	46–80	34–74	90–147

### Results

#### Accuracy

Mean proportion correct values for each group under each condition (set size and distractor congruency) were calculated (see Table 6). Mixed ANOVA revealed no significant main effects or interactions (all *p* values >.09). The accuracy rates were consistently high (between 86 and 92%) across all conditions for both autistic and non-autistic participants.

**Table 6:** Overall mean median RT (ms) and mean accuracy rates (proportion correct) and standard deviations (SD) for the two groups under congruent (cong.) and incongruent (incong.) distractor conditions at each set size for Experiment 2

		Set size 1		Set size 2		Set size 4		Set size 6	
		cong.	incong.	cong.	incong.	cong.	incong.	cong.	incong.
Autism	RT	808	923	965	1022	1231	1298	1509	1490
	(SD)	176	170	217	188	222	282	353	279
	Accuracy	0.92	0.86	0.92	0.91	0.91	0.91	0.9	0.86
	(SD)	0.08	0.1	0.08	0.07	0.11	0.08	0.1	0.15
Neurotypical	RT	815	905	939	989	1142	1167	1418	1377
	(SD)	224	302	217	261	315	260	317	280
	Accuracy	0.9	0.91	0.89	0.88	0.9	0.92	0.88	0.9
	(SD)	0.09	0.08	0.09	0.12	0.06	0.06	0.11	0.09

#### Reaction time

**Fig. 3:**
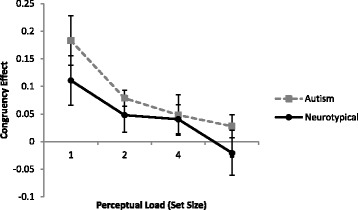
Congruency effects of non-interest items for the two groups across all set sizes. Congruency effects (as a proportion of the baseline RT) were calculated for each person by subtracting average congruent trial RT from the average incongruent RT and dividing by congruent RT. This gives a proportionate measure that accounts for individual differences in RT. *Error bars* indicate the standard error of the mean

The same data reduction steps as used in experiment 1 were applied in the present experiment. Median RT for correct trials in each condition was recorded for each participant (as in many RT studies, the median was used rather than the mean, to avoid extreme values disproportionately influencing the average). A mixed ANOVA was performed using median RT with diagnosis group as the between subjects factor and set size and congruency as within-subject factors. There was a main effect of set size (*F* (3, 81) = 141.8, *p* < .001, η_p_^2^ = 0.84) and congruency (*F* (1, 27) = 15.3, *p* = .001, η_p_^2^ = 0.36) but not of group (*p* > .4). RT increased with set size (set size 1: 862 ms; set size 2: 979 ms; set size 4: 1210 ms; set size 6: 1448 ms) and participants were slower to respond to incongruent trials (congruent 1103 ms; incongruent 1147 ms), but there was no overall difference in RT between the autistic and non-autistic participants. There was a significant interaction between set size and congruency (*F* (3, 81) = 4.0, *p* = .01, η_p_^2^ = 0.13), suggesting that the congruency effect decreased as the load of the task increased (see Fig. 3). No other interactions were significant (all *p* values > .2).

## Discussion

Using selective attention tasks personalized for each participant’s passions and interests, our results show that, for neurotypical individuals, interest and expertise appear to increase the salience of items related to these interests. Given that individuals with autism are widely reported to show patterns of intense interest in certain topics, we had expected them to show increased distraction by images relating to their circumscribed interest—even when performing tasks with high perceptual load. Surprisingly—and in contrast to our initial predictions—we did not see this pattern in the autism group; interest items and non-interest items were treated in the same way: processed at lower levels of load and ignored under higher levels of load. Given our earlier findings of lack of attentional priority for social images [36], this pattern of results supports our second hypothesis outlined above: that there is reduced/absent specialization to any class of stimuli in autism. The inclusion of a self-report measure of interest (PASSION) rules out the possibility that our unexpected result might be due to reduced intensity of interests in our specific group of individuals with autism. It is possible that the two groups had differing levels of insight and consequently accuracy, in their self reports. However, it is not clear how that would impact on the findings (i.e., would autistic people overestimate or underestimate the impact of their interests). It may be interesting in future research to include a parent-report version of the questionnaire. In the self-report version employed here, results showed there was little to differentiate the two groups in any of the subscales within the measure and that both groups reported high levels of interest and expertise. In addition, it should be noted that the current study only involved male participants. It has been shown that circumscribed interests differ in frequency (reduced in females, [56, 57]) and content (random objects/toys rather than mechanical items, [58]) and intensity between males and female and therefore the findings presented here may not reflect the entire autistic population. Subsequent studies with females (autistic and non-autistic) will be important to further explore this area.

This aside, our findings are surprising given that much of the previous literature has found autistic individuals show disproportionate attention to “high autism interest” items, compared to neutral items (e.g., [47–49] as discussed in our introduction above). However, our methods were different from those used in previous studies, allowing us to measure a distinct aspect of attention to items of interest. First, our use of a control group comprised of neurotypical individuals with passions and interests allowed us to examine the qualitative differences between circumscribed interests in autism and typical passions—rather than revealing group differences that may be an artifact of differing prevalence of interests across the two populations. Second, in the current study, we used a selective attention task with competing distractors, rather than a free-viewing paradigm. We were therefore able to demonstrate that the processing of images related to interests and expertise is not automatic and mandatory for autistic people, whereas it is for neurotypical individuals*.* These findings do not necessarily contradict previous findings but build on them: indicating that despite high levels of interest on passive tasks, this is not accompanied by an increase in salience which would lead to automatic priority for attention, even when items of interest compete with the primary task.

Though unexpected, these findings have interesting implications. For example, they are consistent with the body of research that suggests individuals with autism show atypical patterns of neural specialization. Studies have shown that autistic individuals—unlike neurotypical individuals—fail to show activation in the fusiform gyrus during face perception [59, 60], with the patterns of activation more closely resembling the activity observed during feature-based object recognition in the neurotypical population [59]. Altered, reduced, or absent neural specialization for their interests may underlie the lack of automatic attentional priority observed in the present behavioral study.

Secondly, our findings may reflect an altered balance between top-down and bottom-up attentional influences in autism. The Enhanced Perceptual Functioning account of autism suggests that many diagnostic features of autism are due to an increased influence of bottom-up signals on attention relative to that of top-down factors [61]. Indeed, the authors go as far as to suggest that top-down influences are mandatory in neurotypical individuals but optional in autism. Evidence from imaging studies suggests that an increased reliance on bottom-up processes might be responsible for the increased performance found on cognitive tasks such as the block design [62]. This reliance on bottom-up processes is thought to be promoted by a reduction in top-down influences [63].

The possibility of a reduction in top-down modulation of attention has been proposed in autism as a cause of some of the difficulties social and non-social symptoms observed in the condition such as abnormal social gaze [64] and reduced susceptibility to visual illusions. Eye-tracking data obtained by Neumann and colleague (2006) indicates that the increased tendency to focus on the mouth rather than the eyes found in autism occurs despite a lack of group differences in bottom-up attention, suggesting that reduced top-down influences on eye movement are responsible for atypical face processing. This diminished influence of top-down factors is also thought to manifest as some of the non-social aspects of ASD, such as reports of reduced susceptibility to visual illusions in autism [65, 66]. Further support for this theory comes from reports of abnormal influence of categorical knowledge in during a discrimination task, showing that categorical concepts, while still being accessible, exert less of a top-down effect on perception in individuals with autism [67]. These results are analogous with our findings that suggest that circumscribed interests are maintained but do not lead to a top-down influence on attention.

Our results are also in line with recent Bayesian explanations of visual perception in autism, suggesting that a relative bias toward incoming sensory information over prior perceptual experience results in the differences in visual processing reported in the autism literature [68]. In neurotypical individuals, a reduced neural response to repeated, and thus more predictable, stimuli is seen [69, 70]. However, reduced adaptation has been reported in autism during both face perception [71] and gaze detection [71, 72]. Support for this in non-social domains comes from recent findings of reduced repetition suppression being associated with higher levels of autistic traits [73]. Repetition suppression refers to the reduction in the neural response to stimuli when participants are repeatedly exposed to them [74]. Ewbank’s finding indicates that repeated experience to stimuli, as is found in circumscribed interests, has an attenuated effect on the neural responses to these familiar stimuli and provides further evidence for reduced top-down influences on attention in autism. This result has also been linked to some of the clinical symptoms of the condition, such as repetitive behaviors and insistence on sameness [73]. Similarly, it could be these difficulties in the brain’s ability to adapt or habituate to repeated actions or items related to circumscribed interests that lead to such fixations on particular interests. Indeed, it is worth noting that the existence of such a reduction in neural response to repeated stimuli is in line with both reports of increased occurrence of intense circumscribed interests in autism and our present findings that suggest possible reduced neural specialization to stimuli. Therefore, although on first sight counterintuitive, the results we report seem to support a body of the current research and are in keeping with many of the prominent perceptual theories of autism.

It is also important to consider what the practical implications might be of having strong passions that do not appear to change the salience of items related to this interest. Perhaps it results in individuals having to work harder for the same level of reward. For example, in neurotypical attention, items related to interests would capture attention automatically, breaking into awareness as the top-down attention system prioritizes it over other competing items. The reward is gained without the individual needing to actively search for such items. If this top-down system was attenuated or absent then individuals would have to dedicate more effort to interact with items of circumscribed interest for the same reward. This could manifest as the intense levels of interest and an unwillingness to divert from restricted topics that are both characteristic of autistic circumscribed interests. While a full discussion of motivation and cognitive control is beyond the scope of the current paper, this suggestion of a more active dedication of attentional resources to items of interest may resonate with the recent finding that motivational responses in autistic individuals are greater in response to generic “high autism interest” items [75] which in turn leads to less effortful cognitive control in the presence of such items [76]). Further research on a neural level is clearly warranted in order to explore these suggestions; however, for now our behavioral demonstration that autistic circumscribed interests are not accompanied by an increase in attentional salience, offer potential insights into the unique cognitive profile of the condition.

## Conclusions

Overall, our results indicate that while attentional processing in neurotypical individuals is strongly influenced by prior experiences related to their interests, autistic individuals do not show this effect. This is despite the fact that they hold similarly intense interests to the neurotypical individuals tested. We have summarized possible cognitive mechanisms that could explain the disparity observed here. Indeed, taken alongside the literature on the lack of attentional salience for faces in autism, these results may provide preliminary support for atypical neural specialization in autistic individuals.

## Additional files

Additional file 1: Example of PASSION questionnaire. (DOCX 17 kb)

Additional file 2: Raw data from experiment 1.

Additional file 3: Raw data from experiment 2.

Additional file 4: Raw data from the PASSION questionnaire.

## Abbreviations

ADOS: Autism Diagnostic Observation Scale
ASD: Autism Spectrum Disorder
FG: Fusiform gyrus
PASSION: Participant Assessment of Significance of Special Interests and OccupatioNs
RRB: Repetitive behavior, interests, or activities
RT: Response time
SRS: Social Responsiveness Scale
WASI: Wechsler Abbreviated Scale for Intelligence
