# Supplementary material for: A few of my favorite things: circumscribed interests in autism are not accompanied by increased attentional salience on a personalized selective attention task
Source: Mol Autism. 2017 Apr 12;8:20. doi: 10.1186/s13229-017-0132-1 (PMC5389148; doi:10.1186/s13229-017-0132-1)
Supplement: Supplementary file 1 — Example of PASSION questionnaire. (DOCX 17 kb) [file 13229_2017_132_MOESM1_ESM.docx]

Name: Date: Interest/Passion: .

**PASSION: Participant Assessment of Significance of Special Interests and OccupatioNs Remington, Parsons & Bayliss (2015)**

*1. How important is XX to you?* [the most important thing in my life] [extremely important] [very important] [fairly important] [not that important]

*2. How long have you been interested in XX?* [more than 5 years] [3-5 years] [1-3 years] [6 – 12 months] [3-6 months] [less than 3 months]

*3. Are there other interests you have that are as important?* If yes, please list them on the reverse.

*4. I prefer conversations that involve talking about XX* [strongly agree] [agree] [somewhat agree] [somewhat disagree] [disagree] [strongly disagree]

*5a. Most of the conversations I have with my friends involve talking about XX* [strongly agree] [agree] [somewhat agree] [somewhat disagree] [disagree] [strongly disagree]

*5b. Most of the conversations I have with my family involve talking about XX* [strongly agree] [agree] [somewhat agree] [somewhat disagree] [disagree] [strongly disagree]

*5c. Most of the conversations I have with people I’ve just met involve talking about XX* [strongly agree] [agree] [somewhat agree] [somewhat disagree] [disagree] [strongly disagree]

*6. I spend most of my free time doing things that relate to XX* [strongly agree] [agree] [somewhat agree] [somewhat disagree] [disagree] [strongly disagree]

*7. How often do you think about XX:* [several times daily] [daily] [several times per week] [weekly] [several times per month] [monthly] [less than monthly]

*8a. My passion for XX sometimes interferes with my job:* [strongly agree] [agree] [somewhat agree] [somewhat disagree] [disagree] [strongly disagree] [N/A]

*8b. My passion for XX sometimes interferes with my school work:* [strongly agree] [agree] [somewhat agree] [somewhat disagree] [disagree] [strongly disagree] [N/A]

*8c. My passion for XX sometimes interferes with my family relationships:* [strongly agree] [agree] [somewhat agree] [somewhat disagree] [disagree] [strongly disagree] [N/A]

*8d. My passion for XX sometimes interferes with my romantic relationships:* [strongly agree] [agree] [somewhat agree] [somewhat disagree] [disagree] [strongly disagree] [N/A]

*9a. Compared with most people, I know* [a lot more] [a bit more] [about the same] [a bit less] [a lot less] *about XX.*

*9b. Compared with others who are interested in XX, I know* [a lot more] [a bit more] [about the same] [a bit less] [a lot less]
